# Early Laser for Burn Scars (ELABS): protocol for a multi-centre randomised, controlled trial of both the effectiveness and cost-effectiveness of the treatment of hypertrophic burn scars with Pulsed Dye Laser and standard care compared to standard care alone

**DOI:** 10.3310/nihropenres.13234.1

**Published:** 2022-01-18

**Authors:** Mark Brewin, Sharon Docherty, Vanessa Heaslip, Katie Breheny, Jonathon Pleat, Shelley Rhodes

**Affiliations:** 1 Burns & Plastics, Salisbury NHS Foundation Trust, Salisbury, Wiltshire, SP2 8BJ, UK; 2 University of Bournemouth, Poole, Dorset, BH12 5BB, UK; 3 University of Bristol, Bristol, BS8 1QU, UK; 4 Burns & Plastics, North Bristol NHS Trust, Bristol, BS10 5NB, UK; 5 Exeter Clinical Trials Unit, University of Exeter, Exeter, EX4 4SB, UK

**Keywords:** CARe Burn Scale, Early treatment, Health Economic Analysis, Hypertrophic Burn Scars, POSAS, Pulsed Dye Laser (PDL), Quality of Life, RCT

## Abstract

This paper outlines the protocol for a study that is being carried out at multiple centres across the UK in the next three years. It is a Research for Patient Benefit (RfPB) study funded by the National Institute for Healthcare Research (NIHR). The aim is to assess the effectiveness of treating hypertrophic burns scars with pulsed dye laser (PDL) at an early stage of scar formation. The objective is to improve Quality of Life for the patient by improving both the appearance and quality of burn scarring, as well as reducing its psychological impact.

This is a parallel-arm randomised, controlled trial to compare PDL and standard care against standard care alone. The difference is measured between baseline and six-month follow-up. Recruits are within three months of healing from a burn injury; with wounds showing a defined potential for hypertrophic scarring. A total of 120 patients are recruited in a multi-centre study; with randomisation in a 1:1 allocation to each arm. The treatment arm receives 3 PDL treatments at six-week intervals in addition to standard care, whereas the control arm receives standard care alone. The primary outcome is the patient-rated part of the Patient and Observer Scar Scale (POSAS). Psychological and psycho-social impact is evaluated using the CARe burn scale (UWE, Bristol) and Quality Adjusted Life Years (QALY) is determined using the Short-Form Health Survey (SF-12). The study evaluates both the cost-effectiveness through an economic analysis and the patient-reported experience of the treatment by phone interviews.

Trial registration: ISRCTN14392301 (registered on 14 ^th^ June 2021)

Contact for Public & Scientific Queries: Mark Brewin, sft.elabs@nhs.net

Public Title: Early Laser for Burn Scars (ELABS): a trial of the effectiveness and cost of the treatment of hypertrophic burn scars with laser

Countries of Recruitment: England & Scotland

Protocol Version: v11, October 2021

## Introduction

### Hypertrophic scars

Hypertrophic scars are abnormal scars that are red, raised and firm. They occur following surgery, burns or traumatic wounds, usually within 4 – 8 weeks of injury. Survival rates from burns have improved significantly, particularly for injuries of greater total body surface area ^1^. Burns wounds that have not healed within 21 days have a greater likelihood of developing hypertrophic scarring ^2^. Inherently, these increasing numbers of burn patients with large wounds that heal slowly often live with expansive, life-changing hypertrophic scars. Additionally, younger patients seem more predisposed to this complication with a longer lifetime burden of disease ^3^.

The clinical management, of both the aesthetic appearance and any functional impairment of abnormal scarring, has not changed significantly. Early excision and reconstructive surgery is known to reduce infection ^4^ and, in turn, decreases the rate of scarring, but does not prevent it 5. The residual scarring often needs further treatment to improve the outcome; functionally, aesthetically and psychologically.

### Scar incidence

In the United Kingdom, the admission rate with burns is 0.29 per 1,000 people per annum. It is estimated that annually about 250,000 people with burns present to primary care teams and a further 175,000 patients present to emergency departments each year. A systematic review of the epidemiology of scarring after burn injury found that the prevalence rate is between 32 – 72% ^6^. Potentially, more than 100,000 patients sustain such problematic hypertrophic scarring annually.

### Scar impact

Hypertrophic burn scarring (HBS) impacts burn survivors both physically and psychologically. Physical symptoms include itch or pain leading to problematic management with reduction in joint mobility and loss of sleep. A descriptive study on 100 outpatients with HBS showed impaired quality of life (QoL) ^7^. HBS can become psychologically debilitating by affecting self-esteem and body image ^8^. Psychological studies have highlighted problems associated with physical function, emotion, pain and return to work ^6, 9– 11^. The need to improve outcomes for burns patients with hypertrophic scarring has been described; “The greatest unmet challenge after burn injury” ^12^.

### Standard of care

HBS are difficult to treat. National Institute for Health and Care Excellence (NICE, UK) guidelines do not exist for treatment of these scars. Current standards of care (SoC) are dictated by international consensus from expert panels ^13^ and there is little rigorous evidence.

Topical moisturisation with emollient reduces inflammation within the skin by maintaining hydration. Moisturisation forms the baseline of scar treatment yet there is low level evidence for its benefit ^14^. Silicone gel forms the first line of active treatment. They aid scar healing by sealing in moisture, as scars lose hydration more readily ^15^. Pressure garments are used, though sparingly. They are shown to be cost ineffective ^16^, though therapeutically effective when used judiciously ^17^. Data regarding their reduction of excessive scarring are controversial ^18^. Some patients are sensitive to silicone, whereas others cannot tolerate pressure garments. A recent paper showed that these treatments have similar efficacy either used singularly, or in combination ^19^. There appears to be no general consensus as to the optimal treatment regime ^13, 20, 21^. Therefore, there remains an accepted degree of variability in SoC globally.

### Laser treatment

Treatment of burn scars with pulsed dye laser (PDL) is based on anecdotal and case-control study evidence. PDL actively improves scars by destruction of small blood vessels. Reduction of vascularity reduces inflammation that drives the formation of HBS. Conventional scar treatments do not work in this way.

Burn care is performed at a network of National Health Service (NHS, UK) hospitals, where specialised care is centralised. The National Burn Care Standards (2018) stipulate that laser treatment is provided as an option for burn scar patients ^22^. In summary, if the laser intervention is proved effective, it would be available to all patients across the UK burns network.

Research indicates that PDL reduces redness and itch, as well as improving pliability, thickness and texture of the scar ^23– 26^. Laser treatment during early scar formation, or the inflammatory stage, could further improve scarring ^27– 29^. This has been consolidated by a peer-reviewed, published literature review, as performed by the Chief Investigator (CI) ^30^ and a recent systematic review that advocates early intervention with laser ^31^. Early scar intervention is recommended by international experts ^32^; their premise being that it is easier to prevent scar formation than to treat it once it has matured.

There is a paucity of gold-standard, randomised controlled trial (RCT) proof for any treatments of HBS; with the exception perhaps of silicone gel sheeting ^33– 35^. There is, at present, retrospective data that suggest the effectiveness of PDL for the treatment of HBS; but no definitive RCT. The literature also recognises that there is an international push to prevent, rather than treat, HBS and this translates as early intervention ^36– 39^. There is widespread global use of PDL for the treatment of HBS, yet no apparent consensus as to both the parameters and protocol.

There is an unresolved, parallel issue as to whether ablative fractionated laser (AFL), particularly Carbon Dioxide laser, should be studied as the intervention. There are obvious differences between PDL and AFL in basic parameters such as wavelength and energy (or power). These consequently determine both their mechanism of tissue interaction and their effects on scar symptoms and morphology. These two different types of laser are postulated to work in different, but complementary, ways on HBS.

The vascularity of HBS is increased and consists of relatively larger vessels ^40^. One theory is that this aberrant vasculature provides a ready route for successive waves of inflammatory cells to enter the early scar. PDL targets this vasculature specifically with relatively little collateral injury. In doing so, it is suggested that PDL is effective for reduction of both scar redness and itch but moreover, it may limit the tendency for late fibroblast ingress and proliferation with the production of localised, excessive scar tissue, such as collagen. Simplistically, PDL devices may be preventative of later increases in scar thickness and density.

AFL works inherently to destroy tissue by vaporisation in a highly localised spatial orientation. In doing so, they reduce bulk, thickness and stiffness of scars. Early HBS may not have these facets and as such, there is still debate about the utility of AFL devices early in HBS maturation. Moreover, the relatively greater energy levels associated with AFLs may cause more collateral injury including immediate inflammation ^41^ and be associated with more ‘down time’ for the patient.

There are several notable papers that acknowledge that each has their merit and suggested algorithms for laser treatment of burn scars may include both; but with an emphasis seemingly of choosing PDL as the first line ^42– 45^. The authors acknowledge that future algorithms for laser treatment of more problematic scars might involve the AFL devices, particularly where there are significant issues with thickness, density or pliability. The authors also note that there is a concurrent study for the use of Carbon Dioxide AFL for the treatment of HBS in the UK that is being funded by the Scar Free Foundation.

Prior to the evolution of carbon dioxide AFL, a high-quality systematic review stated that “the PDL 595 nm is promising, although more research is necessary. Future research, with a low risk of bias, well-defined scar characteristics, validated outcome measures, standardized measurement methods, follow-up periods of at least 6 months and well-defined laser settings, is needed” ^46^.

In summary, the indications for, and contraindications to, PDL and AFL devices for HBS are yet to be determined conclusively in an RCT; both may have applicability in different settings, but they are unlikely to be interchangeable in clinical utility. The authors feel that PDL needs to be investigated as a relative priority as it has been established for longer, is more widely available and is better tolerated, on average, compared to AFL devices.

There needs to be due consideration of what constitutes an improvement in scar outcome; this study particularly focuses on the outcome for the patient, as opposed to those that the clinician perceives to be important. The PDL has been shown to be an effective treatment for intense pruritus that is common to burn scars ^47^ and it inherently targets redness by destruction of microvessels through selective photothermolysis ^48^. Pruritus has been shown to have strong impact on QoL ^49^ and any effective treatment would therefore be beneficial to the patient. Reduction of redness makes the scarring less obvious and therefore has a positive impact on its psychosocial effect for the patient ^50^. Colour is particularly important as the human eye discriminates colour mismatch more readily than textural abnormality. Therefore, a reduction in relative redness should make the scar less conspicuous from distance and, in turn, have a more significant impact in self-assessment of debility compared to other spheres of visible change.

## Protocol

### Objectives

***Trial rationale.*** This trial aims to clarify the benefit of PDL treatment of HBS in respect of the impact on both the patient’s QoL and scar quality; treatment effectiveness may reduce the impact of the scar. This may translate as both clinical and cost efficiencies for the NHS through reduction in both follow-up appointments and overall treatment costs. Additionally, there is no consensus as to the optimal laser treatment regimen. Though this study does not compare laser treatment settings, this trial would aim to clarify a PDL treatment protocol that was designed from an extensive literature review. Crucially, due to this uncertainty, PDL is not used as SoC within the NHS despite being widely available.

***Research question.*** Does the treatment of HBS with PDL at an early stage in the scar management pathway, in addition to standard care, improve both scar quality and QoL compared to standard care alone, and is this treatment cost-effective?

***Primary objective***

- To determine whether there is significant difference in patient-rated scar quality at 6 months between patients receiving PDL in addition to standard care and standard care alone.

***Secondary objectives***

- To determine whether there is significant difference between the two groups in:
  ▪ Observer-rated scar quality at 6 weeks, 12 weeks and 6 months
  ▪ Patient-rated scar quality at 6 weeks and 12 weeks
  ▪ Patient-rated QoL at 6 months
  ▪ Objectively measured scar redness at 6 months
  ▪ Patient perception of scar change at 6 months
- To conduct a within-trial economic evaluation of PDL to assess its cost-effectiveness.
- To qualitatively explore patient experience and psychosocial impact.
- To assess adverse effect profile of PDL in treatment of burn scars.

### Trial design

***PICO framework***

- P (Patient) – HBS
- I (Intervention) – PDL + SoC
- C (Comparison) – SoC
- O (Outcome)
  • Primary - Patient- and observer-rated Scar Assessment Scale (POSAS, v 2.0)
  • Secondary – Observer-rated POSAS (v 2.0), QoL scales (CAR ^e^ & SF-12), and scar colour (Colorimeter)
- T (Timing) – Primary and Secondary outcomes assessed at baseline and 6 month follow-up
- S (Study type) – RCT

***Trial type.*** This RCT is a parallel-arm, superiority trial with 1:1 allocation ratio. Each trial centre has scrutinised, and contributed to, the trial design and treatment protocol.

***Early treatment.*** It is proposed that the trial investigates that if scars are exposed to PDL within 3 months of wound healing, then scar formation is curtailed by reduction of the proliferation phase ^27, 28, 51^. This means that the scar is prevented from progression to greater degrees of hypertrophy; in turn leading to an improved scar outcome.

***Patient and Public Involvement (PPI).*** In 2017, a PPI group of 18 people (6M, 12F; mean age: 44 years; range: 16–79) met at Salisbury NHS Foundation Trust to discuss the study and its design. Participants were identified from patients that had received PDL, and/or had burn scars. The session was overseen by an independent facilitator. The design for the trial was presented and questions concerning both the use of PDL treatment and trial design were discussed. The group unanimously agreed that the proposed research was necessary, as all patients that received PDL for burn scars saw an obvious added benefit over standard care.

Firstly, the trial design of the RCT was discussed. Options for the treatment vs. control groups were presented:

- early- vs. late-laser
- laser + SoC vs. SoC
- strong vs. weak laser dose
- inter-scar model
- intra-scar model

The group voted for early PDL intervention versus an elective waiting list control group (“late” group) as the most popular trial design (10/18), followed by the SoC option (8/18). Sham laser treatment was deemed non-viable as the sensation of treatment is like “being flicked by an elastic band” and would be hard to replicate. The PPI group was happy that participants in the control arm would have delayed treatment at 6 months, as both PDL is shown to be effective where redness persists ^24^ and it reflects current clinical waiting times. Finally, the PPI group stated that a QoL measure should be used to assess how the patients perceive their scar outcomes. The group discussed that this trial may not include younger children due to the need for general anaesthesia during laser treatment. It was explained that scars have similar characteristics at all ages, so the results of this trial would apply to both adults and children.

A unanimous consensus was obtained that PDL treatment:

- was convenient.
- was relatively painless.
- should become SoC on the basis of their experience, but understood that proposed research was necessary to ensure that their perception was underpinned by rigorous evidence.
- had proved beneficial to all PPI delegates with burn scars.
- may merit the establishment of NICE guidelines for treatment of burn scars.

***Choice of control.*** A recent study recognises that a blinded RCT remains necessary to prove the effectiveness of PDL ^52^. Two previous RCTs have adopted an intra-scar model, which is open to criticism, as the laser may affect the control area due to its contiguity. Also, two areas of scar in close proximity may differ in, for example, relative thickness and vascularity, leading to a biased comparison. Other criticisms of previous studies include inadequate sample size and low laser energy. Similarly, an inter-scar model is difficult as anatomical location can affect the propensity for scarring. Therefore if the scars being compared were at different anatomical sites, this would be a confounding factor.

During study design, the use of sham treatment in the control arm was considered, so that participants are blinded to group allocation and would complete questionnaires without knowing their allocation. The study team concluded that this was not going to be credible for the following reasons:

- A sham laser treatment was considered both using either lower or no output; neither of which would replicate the skin sensation of laser treatment.
- Skin response from laser would feel warm and leads to bruising, which would be obvious to any participant, both in terms of tenderness and appearance.
- There are clear ethical concerns regarding inflicting bruising on participants in the control arm.
- It is imperative that the PIS include treatment side effects; so all participants are aware that laser treatment is felt and results in bruising.
- The PPI group felt that sham laser treatment was deemed non-viable as the sensation of treatment is like “being flicked by an elastic band” and would be hard to replicate.

This choice of control as standard care follows recommendation from a recent feasibility RCT conducted in the UK ^53^ and a consensus between co-applicants for this trial. In a rigorous RCT of the effects of ablative laser on scarring, SoC treatments were chosen as the control ^54^.

***Follow-up duration.*** Though most sites involved in this trial currently use PDL treatment for HBS, some of them are only beginning to develop this service. Indeed, there are many burns services outside the trial sites that do not use PDL at all.

The emphasis from burns and plastics training in the past has been put on treatment rather than prevention of HBS. PDL treatment has conventionally been implemented once the scar has matured significantly. Audits showed that treatment with PDL generally starts at 6 months after healing, though can be delayed up to a year or more if scar formation is delayed. Active scar management, and therefore any scar trial, begins once the wound is healed and there is no longer any need for dressings. Audits of clinic waiting times and standard practice at trial sites show that other active treatments, such as silicone gel or pressure garments, would start in advance of laser treatment. This trial protocol would start laser treatment in line with the development of redness or inflammation within 3 months of the wound healing.

Hypertrophic burn scars proliferate between 1 – 12 months after injury; peaking in growth at around 6 months ^55^. It is also seen that burn scar quality remains constant or deteriorates up to 6 months after injury ^56, 57^. It is proposed that any improvement in POSAS scores at 6 month follow-up may indicate long-term effectiveness. Therefore, for this trial, a follow-up duration of 6 months has been chosen. If the minimum of 3 month criterion for recruitment is considered, allocation occurs at approximately 1 – 3 months after injury; follow-up (or end of participation) is at 7 – 9 months after injury.

As participants in the control arm will be offered laser treatment once their trial participation ends, this may imply a slight delay to any laser treatment for participants in the control arm. However, this choice of follow-up duration makes the trial more attractive to the recruit as each arm may receive laser treatment either at allocation or after approximately 6 months. The trial would further limit the duration after healing by recruiting participants at the earliest stage.

The PPI group suggested that they would be willing to wait at least 6 months for laser intervention but would not be willing to forego treatment entirely. There are ethical concerns regarding completely depriving patients of PDL as this is part of “standard” burns care at some sites. Similarly, recruitment to the trial would be difficult if the alternative to not being in the trial was to be given PDL treatment.

Finally, there is the issue of loss of patients to follow-up as a result of waning compliance if they have a positive result with PDL intervention; attrition of patients in longitudinal burns studies is a common issue which can confound trials ^58^. The authors have allowed for attrition in our sample size calculation.

Standard interval between treatment sessions for PDL is usually 4 – 6 weeks. The bruising resulting from the treatment subsides within 2 – 3 weeks and the effect of the treatment is evident at this point. For this trial, there is around 3 months between the last treatment and measurement of outcome at the primary end-point. This primary endpoint therefore allows a reasonable duration of time for laser treatment to have an observed effect.

It is acknowledged that this is a relatively short follow-up period given that the duration of scar maturation can take upwards of 2 years. A longer-term outcome than 6 months would prove interesting, but the authors hypothesise that a shorter-term improvement would serve to “flatten the curve” of scar maturation, expedite the scarring process and eventually lead to an improved scar outcome. Even at this time point, this may translate as a clinical efficiency and saving for the NHS by a reduction in follow-up appointments and cost of further scar revision; thus justifying the study from the perspective of clinical efficiency.

The 6 month follow-up strikes a compromise between reliable data on scar outcome and a suitable residual size of cohort from which to draw reliable conclusions. It is also a compromise between ensuring a reasonably long follow-up for treatment to show effect and having a research design that was acceptable to potential participants. It is concluded that having a 6 month follow-up is long enough to show any benefits of PDL, but short enough to counter ethical and recruitment concerns.

### Ethics approval and registration

HRA and Health and Care Research Wales (HCRW) Approval has been given for this study, on the basis described in the application form, protocol, supporting documentation and any clarifications received (REC reference 21/SW/0049 and IRAS project ID is 283345). This ethics approval applies to all sites involved in the study. Amendments will be managed using the IRAS platform and disseminated to Principal Investigators at each site by the Study Coordinator. This RCT was also registered with an International Standard Randomised Controlled Trials Number on 14 ^th^ June 2021: https://www.isrctn.com/ISRCTN14392301

## Methods

### Study setting

The participants will be recruited in a national multi-centre trial across NHS settings across England.

The centres involved in this trial will include:

- Salisbury NHS Foundation Trust (CI & Sponsor site)
- Chelsea & Westminster NHS Foundation Trust
- Mid & South Essex Hospital Services NHS Trust
- Newcastle upon Tyne Hospitals NHS Foundation Trust
- North Bristol NHS Trust
- University Hospitals Birmingham NHS Foundation Trust
- With potential addition of a further site at either Leeds or in Scotland.

Each of these sites has been selected as they are part of the UK National Burns Network, where burns care has been centralised. This set of centres captures both the urban and rural population. Multiple sites will increase both recruitment and diversity of the study population and aid generalisability and/or external validity of the study. Site Initiation Visits and standard operating procedures (SOPs) for the trial will ensure consistency across the sites.

Salisbury, Chelsea, Mid & South Essex, Bristol and Birmingham already have laser services set up for scar management; Leeds and Newcastle have operational laser services but do not currently treat scars routinely. PDL treatment is performed at all centres. No additional training is required for laser operators.

### Eligibility criteria

***Inclusion criteria***

NHS patients, with burn injuries >1% Total Body Surface Area, are eligible if they have been treated with skin grafts or had conservatively managed burn wounds or donor sites that:

- have delayed healing of greater than 14 days.
- have potential for hypertrophic scarring.
- are suitable for scar management therapy.

The scar must be within 3 months of healing, where healing time-point is defined by the Healthcare Professional (HCP) during wound management. This time to healing must be for the study scar that is included in the study. The combination of excessive redness with increased thickness and/or hardness provides clear indication of potential scarring, as defined by HCP assessing for scar management.

Where there are multiple scars, the scar having the greatest impact, as reported by the patient, will be included in the trial. Only one scar per patient will be included, and this scar will be identified prior to randomisation. No other scars will be treated with laser until after the study ends for the participant (i.e. post week 26 visit).

The approximate size of the study scar would be anything up to 200 cm ^2^. The size or extent of laser treatment would be determined by or in discussion with the laser operator. The minimum size for the study scar would be 4 cm × 4 cm. The latter is the size of the scar region of interest (ROI) that is used for the colorimeter assessment. In order to ensure the same scar is being treated/assessed on each occasion, the scar will be identified and logged using clinical photography and skin markers, where present.

***Exclusion criteria***

Participant is **NOT** considered eligible for inclusion, if they are:

- unable to give written informed consent.
- below 16 years of age.
- prone to keloid scarring by virtue of either a personal or family history.

Children aged 16 – 18 are able to participate with appropriate consent. Children under 16 would require the added risk of general anaesthesia ^59^. Keloids are different to hypertrophic scars and show only a minimal response to PDL treatment ^60^.

***Other considerations.*** Darker Fitzpatrick skin types (V-VI) with more melanin may reduce effectiveness of the treatment and increase risk of adverse effects, such as permanent pigmentation changes ^61^. They will be included in the trial, but extra information will be made available in the consent process concerning this increased risk. Precautions would be afforded for laser treatment by increasing the pulse duration. Their inclusion will increase global generalisability and recent literature shows good results for these skin types ^45, 62^. Patients with difficulty in understanding English will be provided with translator services.

### Sample size

No published data was found on the Minimal Clinically Important Difference for POSAS. A service evaluation study at Salisbury NHS Trust, on 15 patients treated with PDL, showed a change in the patient-rated POSAS from a mean (SD) of 35.8 (10.6) at baseline to 25.7 (11.2) at 12 months. A study in Holland on the effectiveness of silicone treatment on 46 scars from 23 patients showed mean (SD) pre-treatment scores of 31.0 (7.8) and post-treatment scores of 17.4 (11.5) ^63^. The mean improvement at 12 months was 10.1 in Salisbury and 13.6 in Holland, and pooled over both studies and time-points the SD was around 10. A one-point improvement on each of the 6 items would equate to an overall change of 6 points over the duration of the study of 6 months, which constitutes 11% of the range of the scale (minimum 6, maximum 60). The PPI group felt that a change of 6 points represented an important improvement in scar quality.

A 1:1 allocation ratio is chosen. This design would require 60 participants in the control arm and 60 in the treatment arm to give 90% power; assuming a 2-sided 5% significance level, standard deviation of 10 and effect size of 6. This gives a total of 120 participants and, allowing for 20% drop out, implies recruitment of 150 participants.

This recruitment of 150 patients over 21 months across 7 centres represents 1 per centre per month and is similar to that identified for adults in the PEGASUS trial ^53^. This sample size calculation does not take into account adjustment for baseline POSAS in the analysis. The PEGASUS study found a correlation of 0.545 between baseline and 6 month follow-up POSAS scores ^53^. Taking into account a similar correlation in our study of 150 participants would imply that effect size of 5 (standardised effect size of 0.5) could be detected with 90% power.

The patient-rated POSAS has been chosen as the primary outcome in preference to the observer rated POSAS. The observer-rated POSAS data, scaled similarly, collected in Salisbury and Holland gave a pooled SD of 6.7. For this secondary outcome measure, for which the rater would be blinded as to treatment allocation, the study will have over 99% power to detect a difference of 6 or more between trial arms.

### Recruitment

The study will recruit study participants across 7 sites, with the support of Clinical Research Network (CRN) funded research nurses. The involvement of the CRN research nurses may vary from site to site. For example, a site where CRN research nurses are already screening, approaching and consenting burns patients are very likely to ask the research nurses to screen, approach and consent patients into this study too. A site that is not research active may ask for research nurse support for these activities. Or, alternatively, the PI for that site may feel that, because they see the patients anyway, it is easiest for the PI to screen, approach and consent. The authors acknowledge that there will be a mix of approaches.

Each recruit is allocated a unique code that pseudonymises their data. At each visit, the identification of the participant is verified.

***Recruitment feasibility.*** An online feasibility survey conducted by the CI showed that each study centre treats 30 – 80 patients per year that are eligible for recruitment and fulfil the study criteria. Audits of scar clinic attendance for 2018 show the total possible participants ( Table 1).

**Table 1.** Potential recruitment rates at each site in the trial.

Centre	Per annum	Over recruitment period (21 months)
Salisbury (CI site)	30	52
Chelsea & Westminster	50	88
Leeds/Scotland	45	79
Mid & South Essex	50	88
Newcastle	50	88
UH Birmingham	80	140
North Bristol	50	88
**TOTAL**	**355**	**621**
*East Grinstead*	*50*	*88*
***TOTAL with added sites***	***405***	***709***

This amounts to approximately 621 potential recruits across 7 sites; and with additional sites, if recruitment rate is low, 790 potential recruits across the 9 sites during the 21 month recruitment period. This translates to a recruitment rate of 24% and 19%, respectively, to get the target of 150 recruits.

The uptake to this study, as well as a reduced dropout rate, is favourable for the following reasons:

- Participants are within an NHS pathway with no extra hospital visits required.
- Study design reduces participant burden by predominantly using outcome measures routinely collected.
- PDL treatment is quick and performed as an outpatient.
- PPI group suggested that patients are willing to try new treatment regimens to improve the scar.
- Participants in the control arm would still be offered an option of PDL treatment at the standard time-point of 6 months after randomisation. This would also apply to patients who do not opt into the study.

Recruitment will be reviewed by the Trial Management Group monthly meetings and Trial Steering Committee meetings at approximately 4, 8, 12 & 16 months into the recruitment phase. If recruitment numbers are low, the additional sites may be activated in a change to the protocol.

### Allocation

***Sequence generation.*** Allocation will be determined using a validated password-protected web-based system hosted by the UKCRC registered Clinical Trials Unit (ExeCTU). Randomisation ratio is 1:1 control to treatment and is stratified by study site. The system uses random permuted blocks of varying size, within strata with possible block sizes of 2, 4 or 6.

***Concealment mechanism.*** At first visit, data required for randomisation is entered onto the system. Blinded personnel may enter data for randomisation as the allocation is not displayed on the screen and is only transmitted by e-mail to those specified. The treating healthcare professional will be informed of patient treatment allocation by the CTU via email to their @nhs.net account. The principal investigator at that site will be copied in to this email defining the allocation. Blinded personnel entering data will not receive this e-mail, but will be informed by the system that the randomisation has been successful and that the e-mail has been sent.

***Implementation.*** Randomisation can be performed by all site staff as the result is not displayed to the person inputting the data. The site staff can randomise participants in any order. However, it is expected that it will be done sequentially in chronological order of participant recruitment. A screening log will be collected at site recording all participants that were either recruited or were screened, but were not deemed eligible and why. Details of screening fails will not be entered on the web-based system, but will be collated for the Consolidated Standards of Reporting Trials (CONSORT) Statement. The allocation list will be stored by CTU and access to this list will be managed so that it is not shared with those enrolling and assigning participants.

### Participant timeline

Each participant attends the site for assessment at four study sessions and the primary end-point is at 6 months follow-up ( Table 2).

**Table 2.** Participant timeline over the course of the study.

	STUDY PERIOD					
	Enrolment	Allocation	Post-allocation			Close-out
TIMEPOINT	*-t _1_*	0	*0 weeks*	*6 weeks*	*12 weeks*	*26 weeks*
**ENROLMENT:**						
Eligibility screen	X					
Informed consent	X					
Patient Demographics & Burn History	X					
Allocation		X				
**INTERVENTIONS:**						
PDL(x) + Standard Care			x x x 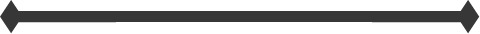			
Standard Care			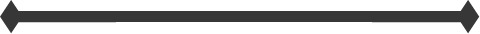			
**ASSESSMENTS:**						
POSAS			X	X	X	X
CAR ^e^ & SF-12			X			X
Colour Measurement			X			X
Healthcare resource use			X			X
Patient Perception Question						X
Qualitative Interview (selected participants)						X

***Baseline data.*** Participant demographic data will be collected at baseline. Skin type will use the standard sixteen group classification of ethnicity currently used in the NHS; in addition to the Fitzpatrick scale. Age and sex will be collected. Additionally the following will be recorded: the depth of burn (superficial, superficial dermal (superficial partial), deep dermal (deep partial), full thickness or mixed); anatomical location; Total Burn Surface Area; aetiology of the burn injury; and time taken to heal for the wound selected for inclusion to the study. These data are all known to affect the likelihood of scarring.

### Outcomes

***Primary outcome.*** Scar Quality - Patient-rated Patient Observer Scar Assessment Scale (POSAS version 2) score at 6 months (mean change from baseline).

***Secondary outcome***

1. Scar Quality: Observer-rated (blinded to treatment allocation) POSAS score at 6 weeks, 12 weeks and 6 months
  Scar Quality: Patient-rated POSAS score at 6 weeks and 12 weeks
2. Quality of Life (QoL) - CARe scale with 14 subscales
3. Incremental cost per Quality Adjusted Life Years (QALY) over 6 months – calculated using Short form Health Survey (SF-12)
4. Healthcare resource use over 6 month follow-up.
5. Scar Redness – Erythema index (0-100) measured by Colorimeter (DSMIII ColorMeter, Cortex Technology, Denmark) at 6 months.
6. Patient’s perception of change from baseline at 6 months - A 7-point scale question evaluates the overall improvement at the primary end-point, as judged by the participant.

***Outcome measures.*** Scar quality and all measures and questionnaires assessed by the patient is for the study scar as a whole. Additionally, Observer POSAS will be performed for the study scar as a whole. Only the colorimeter measurement is carried out for the Scar ROI.

Burn scar quality is assessed using POSAS ^64^; the most commonly used scar assessment scale ^65^ and the only one including the patient’s perception of the scarring. POSAS v2.0 has been shown to be a reliable and feasible tool for the evaluation of scars ^66, 67^. It consists of two parts: a Patient Scale and an Observer Scale. Both scales contain six items that are scored numerically on a ten-step scale (1 – 10). Each makes up a ‘Total Score’, which ranges from 6 – 60 where a higher score reflects reduced scar quality. The mean POSAS scores for scar quality at the primary endpoint of 6 months are compared between trial arms. A reduction in the POSAS score determines whether the proposed intervention improves the scar quality.

The impact on the scarring on the psychological well-being of the patient is assessed using the CARe scale ^68^. The Adult Scale consists of 53 items which cover 14 individual scales; 12 of which fulfilled Rasch and traditional psychometric analyses. These will be analysed in accordance with guidance provided by the CARe team.

QoL is further assessed by SF-12 in order to calculate Quality Adjusted Life Years (QALY). The SF-12 is a multi-purpose short form version of the SF-36 and s a generic measure of health status. The SF-12 physical (PCS-12) is scored using norm-based methods. The scale is transformed to have a mean of 50 and standard deviation of 10.

The resource implications of PDL will be captured in two ways. Secondary care resource use (e.g. clinic visits, resource requirements for PDL delivery) will be collected using a pro forma within the trial case report form (CRF). Primary care resource use (e.g. GP visits) and additional personal expenditures (e.g. burn care products, travel) will be collected using a patient completed questionnaire. This will be based on questionnaires used in previous clinical trials and adapted for the specific study requirements. Secondary care resource use is collected from routine clinic data. Wider costs, such as primary and social care resource use, are collected using direct patient self-reports. Patient-incurred costs are collected at baseline and follow-up.

As the proposed mechanism of PDL is the destruction of micro-vessels within the scar, an objective measurement of vascularity, or redness, is included. Scar colour is measured using the colorimeter (DSM III, Cortex, Denmark), which is a feasible tool that has been validated for the measurement of HBS ^69^. It is important to measure the normal skin to account both for genetic differences between patients and environmentally-related changes in fixed pigmentation (e.g. tanning).

The patient’s perception of change question may correlate any differences seen in scar quality against the overall opinion of the improvement seen by the patient and/or clinician at the primary end-point. It will use a 7-point scale from considerably worse to considerably better. The question is, “What do you think is the overall change to your scar?”

### Data collection

***Blinding (masking).*** Blinded assessments of observer-rated POSAS and colour measurement are performed by scar management HCPs prior to ± laser treatment. The participants are instructed not to divulge their allocation to HCP to maintain blinding. This means that both the observer-rated POSAS and colour measurement are conducted by someone blinded to treatment allocation.

The patient-rated POSAS (primary outcome) and the relevant QoL questionnaires are completed by the participant. Although this assessment cannot be blinded, it was felt, as informed by the PPI group, that the participant’s perspective should be paramount in this trial. It is accepted that this may bias the patient-rated POSAS result in that they will make their rating knowing to which treatment arm they had been allocated. However, blinded clinician-rated secondary outcomes are included. In addition, the data collection, statistical analysis and initial interpretation of the primary and secondary outcome results will be blinded.

***Harms.*** The most serious adverse event in the use of a laser is retinal burn, which may lead to permanent blindness. All laser clinics/sites are subject to government guidelines. The Health and Safety at Work Act 1974 and the Management of Health and Safety at Work Regulations 1999 require the employer and employee to undertake reasonable and practical health and safety measures. This serves to minimise this risk though designation of a “Laser Controlled area”, as defined by the Control of Artificial Optical Radiation at Work Regulations (AOR) 2010, performance of a risk assessment, publication of local rules and use of Personal Protective Equipment. There are no necessary regulatory approvals. All laser sites in the study have appropriate laser safety precautions in place, as determined by the local Laser Protection Advisors.

The adverse events from PDL treatment include, but are not limited to:

•      Blisters

•      Scabbing

•      Excessive oedema

•      Excessive pigmentation changes

•      Excessive Pain

•      Worsening of the scar

•      Other

The scar might be fragile at this early stage but blistering is unlikely. Redness and/or oedema are to be expected and usually resolve within 7 – 10 days. Changes in pigmentation are often caused by the burn injury and the subsequent scarring; hypopigmentation is often inevitable in burn scarring. Hyper-pigmentation resolves or can be treated at a later stage using Q-switched lasers. There have been few documented occurrences of worsening of the scar using PDL.

***Data collection methods***

*Scar assessment*

At each assessment, the participant acclimatises for at least 20 minutes to allow blood flow and skin temperature to equilibrate. The participant remains seated for the duration of assessment. Where the participant has multiple, non-contiguous scars at different anatomical locations, the “worst” scar, as identified by the participant, is assessed and included in the study. It would be prudent to be mindful to avoid areas where they may be a future need for surgery such as areas where contractures are beginning to form.

Whichever treatment group the patient is assigned to, each time they return for assessment, **the same area of hypertrophic scarring must be assessed and treated.** This will be known as the ‘Study Scar’. The ‘Study Scar’ is the scar that the patient, and the designated research team member at weeks 0 and 26, must assess and consider for all their visits. It is important that the participant is aware of this.

The ‘Study Scar’ and each ROI must be selected at the enrolment appointment. Firstly, the scar to be treated and assessed must be identified as a whole; this will be known as the ‘Study Scar’. This will be chosen in agreement with the participant. Where the participant has multiple, non-contiguous scars at different anatomical locations, the ‘Study Scar’ is agreed and included in the study. It would be prudent to be mindful to avoid any area for the ‘Study Scar’ where they may be a future need for surgery such as areas where contractures are beginning to form. The ‘Study Scar’ can be up to about 200 cm ^2^ in size. This limit has been determined as the conventional total area of burn scar that would normally be treated by laser in one visit. Regardless of study group, the limit or guidance for size of ‘Study Scar’ area is the same.

Only part of this hypertrophic scar must then be selected as the ‘Scar ROI’. This should be the part of the burn scar that is the ****most problematic to the participant.**** This is typically excessively red, thickened and firm to touch compared to normal, unburnt skin. An area 4 cm x 4 cm is the optimal size for selection. This will the scar region of interest or ‘Scar ROI’ and this is the area that will be used for the **ColorMeter measurements only**.

The ‘Comparison ROI’ is then identified. It should be an unburnt area of skin. This should be contralateral i.e. the corresponding area on the opposite side of the body. If that contralateral area is burnt also, then the most proximal area to the ‘Scar ROI’ should be identified. This is then drawn onto the ‘Comparison ROI’ map. Again, an (matching) area 4 cm x 4 cm is the optimal size.

Photographs are taken at initial assessment to identify the location of the scar and the assessment point. The investigator must be able to precisely relocate the previously measured site as this could be a source of error that could contribute the variability in scar measurements over time. Skin markers, where seen, are included in the image. A measurement sticker is positioned adjacent to the scar for scaling, and a colour wheel is placed in the frame for white balance. Both are integral to each photograph of the neighbouring scar. Photographs are repeated at each assessment.

POSAS is performed as detailed on the website ( www.posas.org). The observer-rated POSAS is conducted by an HCP. The participant is asked to complete the patient POSAS.

Scar colour is measured using the colorimeter (DSM III, Cortex, Denmark). After calibration, measurements are performed 3 times on the area to be treated and 3 times on a representative adjacent, or symmetrically opposite area of normal skin and then averaged. The colorimeter is connected to a PC. The mean and standard deviation values of Erythema and Melanin are calculated in a spreadsheet (Excel) and these data are then transcribed to the Case Report Form.

The entire assessment takes 20 - 30 minutes. All data is sent to an encrypted folder on a password-protected Health and Social Care Network (HSCN - N3 networked) computer.

**QoL and economic evaluation**

The participant will then complete the CARe scale, SF-12 survey and healthcare resource use questionnaires. The CARe scale questionnaire and SF-12 health-related QoL outcome measure are administered at baseline and at 6 months ^70^ and have been chosen due to its potential to capture the mental health impacts of burns scarring. Secondary care resource use is collected from routine clinic data. Wider costs, such as primary and social care resource use, are collected using direct patient self-reports. Patient-incurred costs are also collected at baseline and follow-up, as part of the qualitative interviews.

**Qualitative interviews**

A sample of participants (n=20, 10 in each arm) receive a telephone interview conducted by a qualitative researcher at approximately 26 weeks. An approximately equivalent proportion is selected from each burn location classification variable; (1) head/neck, (2) torso or (3) limbs). A relative proportion of skin type V – VI will be included in this sample, as laser treatment for these participants may have limited effect.

This interview explores:

- Patient experience of treatment options.
- Social and psychological lifestyle implications.
- Expectations, mind-set, confidence, including the perceived views of carers, where appropriate.

Participants are invited to participate in one-to-one interviews as part of the questionnaires presented in the study. The interview selection strategy includes recruiting a broad range of individuals from different genders, ages, clinical sites and severity of the scarring. In addition, the interviews will also elicit Patient Reported Experience Measures (PREM) by capturing the participants’ perceptions of their experience of the process of care.

Interviews are conducted using interview prompt sheets, pre-agreed with the Patient Advisory Group, to ensure consistency of the interview process. The interview commences with an open-ended introductory question “I am interested in hearing more about your recent experience of … (depending upon which treatment they are provided), please can you tell me about this”. Following this there will be follow-up questions related to:

1)      Study and treatment processes.

2))      Impacts of the treatment process and/or scarring on their daily life.

3))      Psychological and psychosocial implications of the scarring and/or treatment.

4))      Work or financial implications of the treatment process and/or scarring.

5))      Any aspect of their treatment/care that could have been different.

6))      Whether they would recommend this service to others and why.

Lastly the interview will conclude with another open-ended question:

*“Is there anything else you would like to tell me about the treatment you have received?”*

*Harms reporting procedures*

All adverse events should be reported to the CI. Depending on the nature of the event, the reporting procedures below should be followed. Any questions concerning adverse event reporting should be directed to the CI in the first instance. Severity of the adverse event should be reported along with the potential causality. It should also be documented as to whether it represents an unexpected or anticipated event.

**Non serious AEs**

All such events, whether expected or not, should be recorded in the participant’s notes.

**Serious AEs**

An SAE form should be completed and sent to the CI within 24 hours. However, relapse and death and hospitalisations for elective treatment of a pre‐existing condition do not need reporting as SAEs. All SAEs should be reported to the REC where, in the opinion of the Chief Investigator, the event was:

a.      ‘related’, i.e. resulted from the administration of any of the research procedures; and

b.      ‘unexpected’, i.e. an event that is not listed above (ELABS Harms) as an expected occurrence

Reports of related and unexpected SAEs should be submitted by the Sponsor within 15 days of the Chief Investigator becoming aware of the event, using the NRES SAE form for non‐IMP studies. Local investigators should report any SAEs as required by the National Research Ethics Committee and/or local Research & Development Office. All SAE’s and AE’s will be reported to TSC. AE reports will be sent out to all PIs following each TSC meeting. Receipt of these reports will be signed for in local Trial File.

### Retention

All attempts will be made to collect all outcome measures on all participants, regardless of whether they adhered to their treatment. Outcome measures are collected at clinic appointments that form part of their usual care and so it is anticipated that retention will be good. The study design has allowed for up to 20% of participants not to supply data within the sample size considerations.

If a participant does not attend their clinic appointment, this will be rearranged within the next couple of weeks. If this does not happen, attempts will be made to collect those outcome measures that can be collected by phone, prioritising the collection of the primary outcome. If it is not possible to collect data then, where possible, the reason why will be recorded to help with populating the CONSORT flow chart.

Participants in the laser arm of the trial will receive three treatment sessions. A record of attendance at treatment sessions will be kept, and whether they actually received the treatment. If they didn’t receive treatment, the reason why will be recorded, where available.

In consideration of the current COVID crisis, further consideration is made as to the conduct of the study. The primary outcome of patient-rated POSAS can be obtained over the phone when the patient is unable to attend. The observer-rated POSAS can be performed using photos; though this is less reliable particularly for evaluation of thickness and pliability. Patient photographs are also not generally of a sufficient standard. Similarly, the healthcare resource data, patient perception question and qualitative interview can be performed by telephone. Most standard care treatments can be managed remotely, whereas the laser treatment and colour measurement would require the attendance of the patient.

### Data management

The data collection tool for this study will be paper CRFs. Data will be entered directly onto the CRFs and considered a source document. The data collection may be conducted by the CRN nurses, the PIs or the HCP involved in the scar assessment. There may be variation between sites, and this will be detailed in the SOPs for each site.

The recruitment sites will store all original signed informed consent forms (model consent form in the *Extended data* ^71^). All data will be entered electronically onto a database by the Trial Coordinator at the sponsor site or at the participating site where the data originated. This database (REDCap Cloud) is managed by the CTU. Original study forms will be entered and kept on file at the participating site. Copies of 10% of completed forms will be sent by post to the trial coordinator in batches for checking. The CTU will produce reports on data completeness, flag issues and supervise the Trial Coordinator.

All data is recorded and kept in project file for quality control and will be monitored by the sponsor through the Trial Coordinator. Personal data, as defined by General Data Protection Regulation, is not disclosed without written consent. All personal information is kept for the duration of the study and archiving period, after which it will be destroyed. Data archiving costs are included.

### Data Monitoring

***Formal committees***

*Trial Steering Committee*

TSC provides overall supervision for, and regular, impartial oversight of, the study. TSC meetings are scheduled to follow shortly after DM(E)C meetings so that reports from that group are considered if appropriate. TSC meets at 2 months to approve the final protocol, 12 months to review study processes and recruitment, 24 months to approve the statistical analysis plan and 33 months to discuss results and dissemination. At 12 months (9 months into the recruitment phase), TSC review study progress and recommend whether to continue based on likelihood of achieving the recruitment target within timescale. The TSC will compose of an independent Chair, a patient representative, a statistician, a scientist with laser expertise, a clinician with scar and laser expertise and a sponsor representative. Minutes of meetings should be retained in the study master file.

*Data Monitoring (and Ethics) Committee (DM(E)C)*

The DM(E)C will monitor the study and make recommendations to the Trial Steering Committee (TSC). The main consideration is the safety, rights and well-being of the trial participants. The DM(E)C establish their terms of reference at their first meeting. Their main roles are to assess any unexpected adverse effects in the trial and consider new evidence on the safety and effectiveness of laser treatment. General issues such as conduct of the research, compliance to the protocol, recruitment rates, and the data integrity/quality will be discussed. They will also consider any reasons to stop or modify the trial. The DMEC will comprise of two statisticians with experience in study design. Minutes of meetings are retained in the study master file.

### Interventions

***Pulsed Dye Laser (treatment arm).*** There are two models of PDL from different manufacturers; Cynergy (Cynosure, Westford, MA, USA) and the Vbeam (Syneron Candela, USA). The settings to be used are:

- 10 mm, spot size
- 0.5 ms, pulse duration
- 5 – 9 J cm ^-2^, energy fluence
- Approx. 10% overlap
- Single pass

Laser treatment follows assessment and is performed by a trained laser operator. Where multiple scars are present, the laser operator treats the study scar. Other non-contiguous scars may be treated within the limit of total treated area deemed suitable. The energy is selected to produce a degree of purpura during treatment without the presence of skin blanching. The skin response is instantaneous and should be dark purple to black. Blanching (or whitening) in the treatment area suggests over-treatment and is to be avoided. If the operator is unsure, a pause of up to a couple of minutes can be adopted to verify the skin response. Once the desired response is observed, the operator should treat the entire trial scar.

Skin cooling is administered during the treatment. Almost all patients cope with the pain without any anaesthesia; neither topical nor local. The PPI group agreed that pain, though sensate, is not an issue. If necessary, topical anaesthesia is offered and recorded, if used. This provides comfort for the participant and minimises collateral damage. Post-treatment photographs are taken after treatment. Cool Aloe Vera gel may be offered to the participant to apply to the treated area after treatment and its application will be recorded. This may help to soothe any discomfort and reduce the residual heat. The treated areas do not require dressing and after-care information is provided. The patient is advised to avoid perfumed cosmetics, any abrasive drying, tight-fitting clothes, and any strenuous exercise or swimming in chlorinated water for 72 hours. This will include the use of any pressure garments.

At subsequent visits, the participants are asked to report on any adverse effects of the treatment. The previous settings should be used to start the treatment but the operator may increase the energy in 0.5 Jcm ^-2^ steps until a similar response to the previous visit is achieved. All laser settings are recorded for each visit.

***Standard care (control arm).*** Both arms of the trial will be given standard care. The choice of standard care for this trial includes; moisturisation and massage up to 2 – 3 times per day (as directed by the HCP; where maintenance of hydration is required) ± silicone gel treatment ± pressure garments, dependent upon scar maturation.

The control arm receives standard care only. The treatment arm receives a course of three PDL treatments at intervals of 6 weeks, in addition to standard care treatment, as detailed in Table 2. All follow-ups allow ± 1 week to allow for clinic administration.

The following treatments are not permitted for the study scar only and will mean that the participant is withdrawn from the study: Fractional ablative laser, micro-needling, scar revision and/or grafting.

### Statistical methods

***POSAS (Version 2).*** Participants are analysed in trial arm they were initially randomised to regardless of whether treatment was completed (intention-to-treat analysis). Significance tests are 2-sided at the 5% level. Patient component of the POSAS scale at 6 months is the primary outcome measure. In the main analysis, mean POSAS scores at 6 months is compared between trial arms using multiple regression with treatment arm, study site (a design variable) and burn location (a stratification variable coded as (1) head/neck, (2) torso or (3) limbs) as factors and baseline patient POSAS and current age as co-variates. A similar approach will be used for secondary outcome analyses.

For the primary outcome, additional analysis will:

1. consider patient POSAS over all 4 time points (baseline, 6 week, 12 week and 6 months) using a multi-level/mixed model.
2. use multiple imputation to assess the impact of missing data.
3. conduct per-protocol and Complier-Average Causal Effect (CACE) analyses based on completion of the 3 laser sessions.

Prior to the completion of data collection, a detailed statistical analysis plan will be developed and signed off by the CI and TSC.

***Additional analyses***

A similar approach will be used for secondary outcome analyses.

*Colour analysis*

The difference in measured value E (erythema) between the tested and normal area is used as the outcome measure ^72^. A reduction in redness value will indicate an improvement to the scar; a surrogate of inflammation. This value can be correlated against the POSAS for colour or redness.

*Economic analysis*

There is paucity of economic evidence for burn scar treatment. A within-trial economic evaluation is conducted to assess the cost-effectiveness of PDL. The primary analysis is cost-utility analysis from NHS and Personal Social Services perspective. An incremental analysis will compare the differences in costs and Quality Adjusted Life Years (QALY) between SoC and PDL. An exploratory analysis using a societal perspective is performed to capture broader impacts of burns treatment. The SF-6D algorithm is applied to calculate QALY gained ^73^. The economic evaluation follows best-practice guidelines.

*Qualitative analysis*

Individual interviews are audio-recorded and then transcribed verbatim. These anonymised transcripts are analysed thematically using the Braun and Clarke process of thematic analysis ^74^. The analysis is inductive; grounded in the participants’ experiences. The process includes familiarising oneself with the data through repeated reading, searching for meanings and patterns within each focus group/interviews by the qualitative researcher. This leads to the identification of initial codes within each of the interviews, and initial themes from reviewing the codes across the whole data set. Following this, a review of themes leads to the finalisation of the qualitative themes. These are shared and explored across the research team and Patient Advisory Group to ensure both cognisance with the raw data and credibility of the analysis process.

***Analysis population and missing data.*** The main analysis will analyse participants in the group to which they were randomised, regardless of the treatment they actually received. No imputation of missing data will be used for the main analyses, but multiple imputation of missing data will be conducted in additional analyses for the primary outcome. Further details of the approach to be used will be described in a detailed statistical analysis plan

### Ancillary and post-trial care

This is an NHS-sponsored research trial. If an individual suffers negligent harm as a result of participating in the trial, NHS indemnity covers NHS staff and those people responsible for conducting the trial who have honorary contracts with the relevant NHS Trust. In the case of non-negligent harm, the NHS is unable to agree in advance to pay compensation, but an ex-gratia payment may be considered in the event of a claim. Any harm to participants arising from the design or management of the research is covered by the NHS Litigation Authority. There are no arrangements for the Sponsor to pay compensation in the event of harm to research participants where no legal liability arises.

## Dissemination

The full study protocol will be published in NIHR Open Research. Results from this study will be disseminated to HCPs through both publications in the journal *Burns* and presentations at British Burns Association (BBA), European Burns Association (EBA) and/or International Society of Burn Injury (ISBI) annual meetings. The main publication will be submitted to *Burns Open* and this is costed in the budget. The main publication will be authored by the Trial Management Group, which consists of the co-applicants and principal investigators. The qualitative component of the RCT is published within a high impact journal such as *BMC (Open)*. The participant level dataset and statistical code will be archived on the BU data repository BORDaR ( https://bordar.bournemouth.ac.uk/)

If the study proves effectiveness of PDL, it could underpin the evidence for NICE guidelines of PDL burn scar treatment; if ineffective, the provision of PDL in the NHS may be revised. The evidence should improve how healthcare is delivered in line with the RfPB statement. The outcome of this study may be generalised for hypertrophic scars from surgery or other trauma.

The treatment protocol will be shared across the UK burns community through the Burns Operational Delivery Networks. Key study findings are relayed to burn scar participants and the wider patient group through lay summaries for burns support forums/websites, such as Dan’s Fund and TalkHealth. Details of the Patient and Public Involvement process and how this has shaped the study are submitted to a journal such as *Research Involvement and Engagement*.

## Study status

As of November 2021, the study is in final set-up stage for some sites and recruitment stage is imminent. The study database has been written on REDCap and is ready for use.

## Roles and responsibilities

**Chief Investigator:** Dr Mark Brewin

**Co-applicants**

•      Position to be filled (Statistician, Bournemouth University Clinical Research Unit, BUCRU)

•      Dr Sharon Docherty (Statistician, BUCRU)

•      Dr Vanessa Heaslip (Associate Professor, Department of Nursing Science, Bournemouth University)

•      Dr Katie Breheny (Health Economist, University of Bristol)

•      Dr Shelley Rhodes (Senior Trial Manager, Exeter CTU, University of Exeter)

•      Mr Jonathon Pleat (Consultant Burns Plastics Surgeon, North Bristol NHS Trust)

•      Kate Attrill (Senior Physiotherapist, Chelsea and Westminster Hospital NHS Foundation Trust)

•      Tara Mack (Surgical Care Practitioner/Laser lead, Mid and South Essex NHS Foundation Trust)

**Contributorship**

MB conceived of the trial. Professor Pete Thomas (Retired, BUCRU), MB, SD, VH, KB & JP initiated the trial design. VH, SR and SD provided statistical expertise in clinical trial design and are conducting both the primary statistical and qualitative analysis. SR is the senior trial manager at the Clinical Trials Unit (CTU) and is involved in data management, randomisation and project oversight. KB is the health economist for the trial. KA is a research therapist and TM is a research nurse. All authors contributed to refinement of the trial protocol and approved the final manuscript.

**Sponsor contact information**

Trial Sponsor: Salisbury NHS Foundation Trust

Contact Name: Dr Stef Scott

Address: R&D Office, Salisbury District Hospital, Odstock Road, Salisbury, Wiltshire, SP2 8BJ

Tel: (01722) 425027

Email: stef.scott1@ nhs.net

**Trial Coordination Centre**

For general queries, supply of trial documentation, and collection of data, please contact:

Trial Coordinator: Ruth Fennelly

Address: R&D Office, Salisbury NHS Foundation Trust, Odstock Road, Salisbury, Wiltshire, SP2 8BJ

Tel: 07570 221971

Email: sft.elabs@nhs.net

**Sponsor and funder**

This funding source had no role in the design of this trial and will not have any role during its execution, analyses, interpretation of the data, or decision to submit results.

- Sponsor services provide would include:
- Supporting site set up – site files, delegation logs etc.
- Support/advice with regulatory approvals including completion of forms (REC, Statement of Activities, amendments)
- Support with development of SIV training material (e.g. admin & site file maintenance)
- Support appointing & training trial coordinator
- Ensuring trial close down and archiving happens in a timely manner

**Chief Investigator:** Dr Mark Brewin

- Design and conduct of ELABS trial
- Preparation of protocol and revisions
- Preparation of patient information sheets (PIS) and CRFs (Case Report Forms)
- Site Initiation visits (SIV)
- Trial registrations (ISRCTN & IRAS)
- Organising steering committee meetings
- Chair and organise PMG (Project Management Group)
- Publication of trial reports

**Committees**

**Project Management Group (PMG)**

Membership: All co-applicants; and PIs by invitation or agreement. CI will chair the PMG.

- Prepare and discuss material for Patient Advisory Group
- All lead investigators and co-applicants will be PMG members.
- Discuss recruitment rates and project time-points
- Discuss and develop patient and public involvement (PPI) input via PPI lead
- Reviewing progress of trial and if necessary agreeing changes to the protocol to facilitate the smooth running of the trial.
- Liaise with Exeter CTU team and Trial Coordinator for data management
- Discuss issues/concerns
- Discuss and review statistical analysis plan and health economics plan
- Discuss and review publication/ dissemination plan

**Trial Steering Committee (TSC)**

Baljit Dheansa (Chair), Chris Bonney (Patient representative), Lizzi Pitt (Statistician), Thomas Lister (Scientist with laser expertise), Ioannis Goutos (Clinician for laser adverse events), Sponsor representative

TSC Responsibilities:

- Agreement of final protocol
- Review study processes and recruitment
- Trial Monitoring
- Data Integrity & Patient Safety
- Approve statistical analysis plan
- Discuss results and dissemination
- Auditing trial conduct

**Data Monitoring (and Ethics) Committee (DM(E)C)**

- Assessment of any unexpected adverse effects in the trial
- Consideration of new evidence on the safety and effectiveness of laser treatment. Discussion of General issues such as data integrity and participant complaints/withdrawals.

## Data availability

### Underlying data

No data are associated with this article.

### Extended data

Figshare: ELABS Consent Form, https://doi.org/10.6084/m9.figshare.16940188 ^71^.

### Reporting guidelines

Figshare: SPIRIT checklist for ‘Early Laser for Burn Scars (ELABS): protocol for a multi-centre randomised, controlled trial of both the effectiveness and cost-effectiveness of the treatment of hypertrophic burn scars with Pulsed Dye Laser and standard care compared to standard care alone’, https://doi.org/10.6084/m9.figshare.16940179.v1 ^75^.

Data are available under the terms of the Creative Commons Zero "No rights reserved" data waiver (CC0 1.0 Public domain dedication).

## References

[1] Brusselaers N Hoste EAJ Monstrey S : Outcome and changes over time in survival following severe burns from 1985 to 2004. *Intensive Care Med.* 2005;31(12):1648–1653. 10.1007/s00134-005-2819-6 16220315

[2] Deitch E Wheelan T Rose M : Hypertrophic burn scars: analysis of variables. *J Trauma.* 1983;23(10):895–8. 6632013

[3] Wallace HJ Fear MW Crowe MM : Identification of factors predicting scar outcome after burn injury in children: a prospective case-control study. *Burns Trauma.* 2017;5(1):19. 10.1186/s41038-017-0084-x 28680887 PMC5494810

[4] Herndon DN Barrow RE Rutan RL : A comparison of conservative versus early excision. Therapies in severely burned patients. *Ann Surg.* 1989;209(5):547–552; discussion 552–3. 10.1097/00000658-198905000-00006 2650643 PMC1494069

[5] Cubison TCS Pape SA Parkhouse N : Evidence for the link between healing time and the development of hypertrophic scars (HTS) in paediatric burns due to scald injury. *Burns.* 2006;32(8):992–999. 10.1016/j.burns.2006.02.007 16901651

[6] Lawrence JW Mason ST Schomer K : Epidemiology and impact of scarring after burn injury: a systematic review of the literature. *J Burn Care Res.* 2012;33(1):136–146. 10.1097/BCR.0b013e3182374452 22138807

[7] Bock O Schmid-Ott G Malewski P : Quality of life of patients with keloid and hypertrophic scarring. *Arch Dermatol Res.* 2006;297(10):433–438. 10.1007/s00403-006-0651-7 16528552

[8] Van Loey NEE Van Son MJM : Psychopathology and psychological problems in patients with burn scars: epidemiology and management. *Am J Clin Dermatol.* 2003;4(4):245–72. 10.2165/00128071-200304040-00004 12680803

[9] Oh H Boo S : Assessment of burn-specific health-related quality of life and patient scar status following burn. *Burns.* 2017;43(7):1479–1485. 10.1016/j.burns.2017.03.023 28539239

[10] Spronk I Polinder S Haagsma JA : Patient-reported scar quality of adults after burn injuries: A five-year multicenter follow-up study: Long-term patient-reported scar quality of adults after burn injuries. *Wound Repair Regen.* 2019;27(4): 406–414. 10.1111/wrr.12709 30793408 PMC6850449

[11] Brewin MP Homer SJ : The lived experience and quality of life with burn scarring-The results from a large-scale online survey. *Burns.* 2018;44(7):1801–1810. 10.1016/j.burns.2018.04.007 30072198

[12] Finnerty CC Jeschke MG Branski LK : Hypertrophic scarring: the greatest unmet challenge after burn injury. *Lancet.* 2016;388(10052):1427–1436. 10.1016/S0140-6736(16)31406-4 27707499 PMC5380137

[13] Monstrey S Middelkoop E Vranckx JJ : Updated scar management practical guidelines: non-invasive and invasive measures. *J Plast Reconstr Aesthet Surg.* 2014;67(8):1017–1025. 10.1016/j.bjps.2014.04.011 24888226

[14] Klotz T Kurmis R Munn Z : Moisturisers in scar management following burn: A survey report. *Burns.* 2017;43(5):965–972. 10.1016/j.burns.2017.01.021 28413108

[15] Momeni M Hafezi F Rahbar H : Effects of silicone gel on burn scars. *Burns.* 2009;35(1):70–74. 10.1016/j.burns.2008.04.011 18672332

[16] Atiyeh BS El Khatib AM Dibo SA : Pressure garment therapy (PGT) of burn scars: evidence-based efficacy. *Ann Burns Fire Disasters.* 2013;26(4):205–212. 24799851 PMC3978593

[17] Engrav LH Heimbach DM Rivara FP : 12-Year within-wound study of the effectiveness of custom pressure garment therapy. *Burns.* 2010;36(7):975–983. 10.1016/j.burns.2010.04.014 20537469

[18] Anzarut A Olson J Singh P : The effectiveness of pressure garment therapy for the prevention of abnormal scarring after burn injury: a meta-analysis. *J Plast Reconstr Aesthet Surg.* 2009;62(1):77–84. 10.1016/j.bjps.2007.10.052 18249046

[19] Wiseman J Simons M Kimble R : Effectiveness of topical silicone gel and pressure garment therapy for burn scar prevention and management in children: study protocol for a randomised controlled trial. *Trials.* 2017;18(1):72. 10.1186/s13063-017-1820-z 28209175 PMC5314463

[20] Friedstat JS Hultman CS : Hypertrophic burn scar management: what does the evidence show? A systematic review of randomized controlled trials. *Ann Plast Surg.* 2014;72(6):S198–S201. 10.1097/SAP.0000000000000103 24835874

[21] Tredget EE Shupp JW Schneider JC : Scar management following burn injury. *J Burn Care Res.* 2017;38(3):146–147. 10.1097/BCR.0000000000000548 28338518 PMC9990899

[22] National Standards for Provision and Outcomes in Adult and Paediatric Burn Care. British Burn Association,2018. Reference Source

[23] Kono T Erçöçen AR Nakazawa H : Treatment of hypertrophic scars using a long-pulsed dye laser with cryogen-spray cooling. *Ann Plast Surg.* 2005;54(5):487–493. 10.1097/01.sap.0000155276.93061.93 15838209

[24] Alster TS Nanni CA : Pulsed dye laser treatment of hypertrophic burn scars. *Plast Reconstr Surg.* 1998;102(6):2190–5. 10.1097/00006534-199811000-00060 9811021

[25] Chan HH Wong DS Ho WS : The use of pulsed dye laser for the prevention and treatment of hypertrophic scars in Chinese persons. *Dermatol Surg.* 2004;30(7):987–994; discussion 994. 10.1111/j.1524-4725.2004.30303.x 15209788

[26] Zuccaro J Ziolkowski N Fish J : A systematic review of the effectiveness of laser therapy for hypertrophic burn scars. *Clin Plast Surg.* 2017;44(4):767–779. 10.1016/j.cps.2017.05.008 28888302

[27] Liew SH Murison MSC Dickson WA : Prophylactic treatment of deep dermal burn scar to prevent hypertrophic scarring using the pulsed dye laser: a preliminary study. *Ann Plast Surg.* 2002;49(5):472–5. 10.1097/00000637-200211000-00005 12439013

[28] McCraw JB McCraw JA McMellin A : Prevention of unfavorable scars using early pulse dye laser treatments: a preliminary report. *Ann Plast Surg.* 1999;42(1):7–14. 10.1097/00000637-199901000-00002 9972711

[29] Behrouz-Pirnia A Liu H Peternel S : Early laser intervention to reduce scar formation in wound healing by primary intention: A systematic review. *J Plast Reconstr Aesthet Surg.* 2020;73(3):528–536. 10.1016/j.bjps.2019.09.050 31757687

[30] Brewin MP Lister TS : Prevention or treatment of hypertrophic burn scarring: A review of when and how to treat with the Pulsed Dye Laser. *Burns.* 2014;40(5):797–804. 10.1016/j.burns.2013.12.017 24439925

[31] Karmisholt KE Haerskjold A Karlsmark T : Early laser intervention to reduce scar formation - a systematic review. *J Eur Acad Dermatol Venereol.* 2018;32(7):1099–1110. 10.1111/jdv.14856 29419914

[32] Gold MH McGuire M Mustoe TA : Updated international clinical recommendations on scar management: part 2 - algorithms for scar prevention and treatment. *Dermatol Surg.* 2014;40(8):825–831. 25068544 10.1111/dsu.0000000000000050

[33] Meaume S Le Pillouer-Prost A Richert B : Management of scars: updated practical guidelines and use of silicones. *Eur J Dermatol.* 2014;24(4):435–43. 10.1684/ejd.2014.2356 25141160

[34] Nedelec S Carter A Forbes L : Practice guidelines for the application of nonsilicone or silicone gels and gel sheets after burn injury. *J Burn Care Res.* 2015;36(3):345–374. 10.1097/BCR.0000000000000124 25094007

[35] Gold MH Berman B Clementoni MT : Updated international clinical recommendations on scar management: part 1 - evaluating the evidence. *Dermatol Surg.* 2014;40(8):817–824. 25068543 10.1111/dsu.0000000000000049

[36] Westra I Pham H Niessen FB : Topical silicone sheet application in the treatment of hypertrophic scars and keloids. *J Clin Aesthet Dermatol.* 2016;9(10):238–35. 27847546 PMC5104309

[37] Alberti LR Vicari EF De Souza Jardim Vicari R : Early use of CO _2_ lasers and silicone gel on surgical scars: Prospective study. *Lasers Surg Med.* 2017;49(6):570–576. 10.1002/lsm.22634 28117496

[38] Bailey JK Blackstone BN DeBruler DM : Effects of early combinatorial treatment of autologous split-thickness skin grafts in red duroc pig model using pulsed dye laser and fractional CO _2_ laser. *Lasers Surg Med.* 2018;50(1):78–87. 10.1002/lsm.22702 28759110

[39] Karmisholt KE Haerskjold A Karlsmark T : Early laser intervention to reduce scar formation - a systematic review. *J Eur Acad Dermatol Venereol.* 2018;32(7):1099–1110. 10.1111/jdv.14856 29419914

[40] Liew Y McLaughlin R Gong P : *In vivo* assessment of human burn scars through automated quantification of vascularity using optical coherence tomography. *J Biomed Opt.* 2012;18(6):061213. 10.1117/1.JBO.18.6.061213 23174911

[41] Azzam OA Bassiouny DA El-Hawary MS : Treatment of hypertrophic scars and keloids by fractional carbon dioxide laser: a clinical, histological, and immunohistochemical study. *Lasers Med Sci.* 2016;31(1):9–18. 10.1007/s10103-015-1824-4 26498451

[42] Gold MH McGuire M Mustoe TA : Updated international clinical recommendations on scar management: part 2--algorithms for scar prevention and treatment. *Dermatol Surg.* 2014;40(8):825–831. 10.1111/dsu.0000000000000050 25068544

[43] Willows BM Ilyas M Sharma A : Laser in the management of burn scars. *Burns.* 2017;43(7):1379–1389. 10.1016/j.burns.2017.07.001 28784339

[44] Anderson RR Donelan MB Hivnor C : Laser treatment of traumatic scars with an emphasis on ablative fractional laser resurfacing: consensus report. *JAMA Dermatol.* 2014;150(2):187–193. 10.1001/jamadermatol.2013.7761 24336931

[45] Zuccaro J Muser I Singh M : Laser therapy for pediatric burn scars: Focusing on a combined treatment approach. *J Burn Care Res.* 2018;39(3):457–462. 10.1093/jbcr/irx008 29897540

[46] Vrijman C van Drooge AM Limpens J : Laser and intense pulsed light therapy for the treatment of hypertrophic scars: a systematic review. *Br J Dermatol.* 2011;165(5):934–42. 10.1111/j.1365-2133.2011.10492.x 21711337

[47] Allison KP Kiernan MN Waters RA : Pulsed dye laser treatment of burn scars. Alleviation or irritation? *Burns.* 2003;29(3):207–213. 10.1016/s0305-4179(02)00280-2 12706612

[48] Anderson RR Parrish JA : Selective photothermolysis: precise microsurgery by selective absorption of pulsed radiation. *Science.* 1983;220(4596):524–7. 10.1126/science.6836297 6836297

[49] Parnell LKS Nedelec B Rachelska G : Assessment of pruritus characteristics and impact on burn survivors. *J Burn Care Res.* 2012;33(3):407–418. 10.1097/BCR.0b013e318239d206 22210065

[50] Tao J Champlain A Weddington C : Treatment of burn scars in Fitzpatrick phototype III patients with a combination of pulsed dye laser and non-ablative fractional resurfacing 1550 nm erbium:glass/1927 nm thulium laser devices. *Scars Burns Heal.* 2018;4:205951311875851. 10.1177/2059513118758510 29799583 PMC5965338

[51] Bloemen MCT van der Veer WM Ulrich MMW : Prevention and curative management of hypertrophic scar formation. *Burns.* 2009;35(4):463–475. 10.1016/j.burns.2008.07.016 18951704

[52] Hultman CS Edkins RE Wu C : Prospective, before-after cohort study to assess the efficacy of laser therapy on hypertrophic burn scars. *Ann Plast Surg.* 2013;70(5):521–6. 10.1097/SAP.0b013e31827eac5e 23542846

[53] Moiemen N Mathers J Jones L : Pressure garment to prevent abnormal scarring after burn injury in adults and children: the PEGASUS feasibility RCT and mixed-methods study. *Health Technol Assess.* 2018;22(36):1–162. 10.3310/hta22360 29947328 PMC6036406

[54] Douglas H Lynch J Harms KA : Carbon dioxide laser treatment in burn-related scarring: A prospective randomised controlled trial. *J Plast Reconstr Aesthet Surg.* 2019;72(6):863–870. 10.1016/j.bjps.2019.01.027 30846294

[55] Gauglitz GG Korting HC Pavicic T : Hypertrophic scarring and keloids: pathomechanisms and current and emerging treatment strategies. *Mol Med.* 2011;17(1–2):113–125. 10.2119/molmed.2009.00153 20927486 PMC3022978

[56] Manuskiatti W Fitzpatrick RE : Treatment response of keloidal and hypertrophic sternotomy scars: comparison among intralesional corticosteroid, 5-fluorouracil, and 585-nm flashlamp-pumped pulsed-dye laser treatments. *Arch Dermatol.* 2002;138(9):1149–55. 10.1001/archderm.138.9.1149 12224975

[57] van der Wal MBA Vloemans JFPM Tuinebreijer WE : Outcome after burns: An observational study on burn scar maturation and predictors for severe scarring. *Wound Rep Regen.* 2012;20(5):676–687. 10.1111/j.1524-475X.2012.00820.x 22985039

[58] Bamer AM McMullen K Gibran N : Factors Associated with Attrition of Adult Participants in a Longitudinal Database: A National Institute on Disability, Independent Living, and Rehabilitation Research Burn Model System Study. *J Burn Care Res.* 2020;41(2):270–279. 10.1093/jbcr/irz186 31738436 PMC9121819

[59] Wong BM Keilman J Zuccaro J : Anesthetic Practices for Laser Rehabilitation of Pediatric Hypertrophic Burn Scars. *J Burn Care Res.* 2017;38(1):e36–e41. 10.1097/BCR.0000000000000427 27532615

[60] Arno AI Gauglitz GG Barret JP : Up-to-date approach to manage keloids and hypertrophic scars: A useful guide. *Burns.* 2014;40(7):1255–1266. 10.1016/j.burns.2014.02.011 24767715 PMC4186912

[61] Tucker DI Aden J Renz EM : Quantitative assessment of pulse dye laser therapy in the management of hypertrophic burn scars. *Ann Clin Lab Res.* 2015;3(4).

[62] Klifto KM Asif M Hultman CS : Laser management of hypertrophic burn scars: a comprehensive review. *Burns Trauma.* 2020;8:tkz002. 10.1093/burnst/tkz002 32346540 PMC7175764

[63] van der Wal MBA van Zuijlen PP van de Ven P : Topical silicone gel versus placebo in promoting the maturation of burn scars: a randomized controlled trial. *Plast Reconstr Surg.* 2010;126(2):524–531. 10.1097/PRS.0b013e3181e09559 20679835

[64] Draaijers LJ Tempelman FRH Botman YAM : The patient and observer scar assessment scale: a reliable and feasible tool for scar evaluation. *Plast Reconstr Surg.* 2004;113(7):1960–5; discussion 1966–7. 10.1097/01.prs.0000122207.28773.56 15253184

[65] Bae SH Bae YC : Analysis of frequency of use of different scar assessment scales based on the scar condition and treatment method. *Arch Plast Surg.* 2014;41(2):111–5. 10.5999/aps.2014.41.2.111 24665417 PMC3961606

[66] van der Wal MBA Tuinebreijer WE Bloemen MCT : Rasch analysis of the Patient and Observer Scar Assessment Scale (POSAS) in burn scars. *Qual Life Res.* 2012;21(1):13–23. 10.1007/s11136-011-9924-5 21598065 PMC3254877

[67] van de Kar AL Corion LUM Smeulders MJC : Reliable and feasible evaluation of linear scars by the Patient and Observer Scar Assessment Scale. *Plast Reconstr Surg.* 2005;116(2):514–22. 10.1097/01.prs.0000172982.43599.d6 16079683

[68] Griffiths C Guest E Pickles T : The Development and Validation of the CARe Burn Scale—Adult Form: A Patient-Reported Outcome Measure (PROM) to Assess Quality of Life for Adults Living with a Burn Injury. *J Burn Care Res.* 2019;40(3):312–326. 10.1093/jbcr/irz021 30820556

[69] van der Wal M Bloemen M Verhaegen P : Objective color measurements: clinimetric performance of three devices on normal skin and scar tissue. *J Burn Care Res.* 2013;34(3):e187–94. 10.1097/BCR.0b013e318264bf7d 23237819

[70] Ware Jr J Kosinski M Keller SD : A 12-Item Short-Form Health Survey: construction of scales and preliminary tests of reliability and validity. *Med Care.* 1996;34(3):220–233. 10.1097/00005650-199603000-00003 8628042

[71] Brewin M : ELABS Consent Form. figshare. Online resource.2021. 10.6084/m9.figshare.16940188.v1

[72] van der Wal M Bloemen M Verhaegen P : Objective color measurements: clinimetric performance of three devices on normal skin and scar tissue. *J Burn Care Res.* 2013;34(3):e187–e194. 10.1097/BCR.0b013e318264bf7d 23237819

[73] Brazier JE Roberts J : The estimation of a preference-based measure of health from the SF-12. *Med Care.* 2004;42(9):851–859. 10.1097/01.mlr.0000135827.18610.0d 15319610

[74] Braun V Clarke V : Using thematic analysis in psychology. *Qual Res Psychol.* 2006;3(2):77–101. 10.1191/1478088706qp063oa

[75] Brewin M : ELABS SPIRIT Checklist. figshare. Online resource.2021. 10.6084/m9.figshare.16940179.v1

