# Supplementary figures and images for: Early Laser for Burn Scars (ELABS): protocol for a multi-centre randomised, controlled trial of both the effectiveness and cost-effectiveness of the treatment of hypertrophic burn scars with Pulsed Dye Laser and standard care compared to standard care alone [version 1; peer review: 2 approved]
Source: NIHR Open Res. Author manuscript; Available in PMC 2022 Apr 6. (PMC7612584)

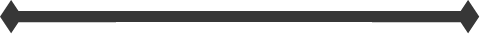

Supplement: Image in table 2 [file EMS143973-supplement-Image_in_table_2.tif]
