# Supplementary material for: Early Laser for Burn Scars (ELABS): protocol for a multi-centre randomised, controlled trial of both the effectiveness and cost-effectiveness of the treatment of hypertrophic burn scars with Pulsed Dye Laser and standard care compared to standard care alone [version 1; peer review: 2 approved]
Source: NIHR Open Res. Author manuscript; Available in PMC 2022 Apr 6. (PMC7612584)
Supplement: Open Peer Review [file EMS143973-supplement-Open_Peer_Review.pdf]

Open Peer Review

Current Peer Review Status: ✔ ✔

Version 1

Reviewer Report 28 February 2022

https://doi.org/10.3310/nihropenres.14350.r28465

© 2022 Shah M. This is an open access peer review report distributed under the terms of the Creative Commons Attribution License, which permits unrestricted use, distribution, and reproduction in any medium, provided the original work is properly cited.

✔ **Mamta Shah**

Burns and Plastic Surgery Department, Royal Manchester Children's Hospital, Manchester, UK

The researchers present a well thought out and reasoned study protocol on the early use of LASER to minimise the morbidity of post-burn hypertrophic scarring. They define early as the period within 3 months of complete healing. Hypertrophic scarring following a burn injury is one of the most common causes of morbidity for burn patients resulting in functional and psychological consequences. There is emergent evidence to demonstrate that the use of PDL on post-burn hypertrophic scars (HBS) alleviates itching and reduces redness of the scars.

The basis of this randomised controlled trial is to ascertain if the use of PDL early in the trajectory of the post-burn hypertrophic scar process, prevents the scar from becoming red, lumpy, firm/hard and causing subsequent problems with function and symptoms such as itching as well as alleviating the existing symptoms and quality of the scar at start of treatment.

The researchers have involved patient group and discussed all aspects of the study with them. However, it is unclear how many of the 18 patients involved in this study design and discussion had already had laser treatment and how many had not? Was there an element of bias in the discussions from the group who had the PDL treatment if not equally distributed?

The researchers have selected a period of within 3 months of healing of target site as a criteria and not from time of injury. That has to be commended as it is important to ensure the trajectory is from time to complete healing and not time of injury! It is also important that the final follow-up assessment is at least 3 months from time of last intervention and not 6 months from time of 1st treatment. Another point to note in patient selection, the researchers are selecting not only post-burn scars but also donor site scars for their study. These different mechanistic scars should not be included into one cohort as a collective due to the different mechanisms of injury and subsequent wound healing and scarring (Jabeen *et. al*; 2019[1]).

The researchers have stated that this study does not test the various PDL treatment settings/intensity and duration but that they have selected their regime from a review of literature: perhaps some clarification on how this was done would be useful. If the various centres

participating in this study are already using PDL for management of their burn patients, how did they agree to the current protocol as the optimum one to use?

The primary and secondary objectives are well defined. However, the final assessment of 6 months i.e. 3 months from final treatment, will not allow the researchers to make any claims of long term impact on the post-burn hypertrophic scarring with regard to alteration of the trajectory or prevention of long term sequelae. They have explained their rationale but it would be prudent to include in their ethics design and study protocol a caveat that allows them to follow these patients long term if we are to get any insight into whether early LASER intervention precludes long term sequelae of HBS and indeed pushes the natural trajectory of scar maturation process to the left. Some or most of these patients, presumably will be followed up in the Consultants clinics and if so, would not be too cumbersome to assess them long term. It would be a shame to lose this opportunity of ensuring the long term impact of early intervention as it is crucial to the study "hypothesis". Furthermore, it will ascertain how long the effects of PDL last if not permanent.

References

1. Jabeen S, Clough ECS, Thomlinson AM, Chadwick SL, et al.: Partial thickness wound: Does mechanism of injury influence healing?. *Burns*. **45** (3): 531-542 PubMed Abstract | Publisher Full Text

Is the rationale for, and objectives of, the study clearly described?

Yes

Is the study design appropriate for the research question?

Yes

Are sufficient details of the methods provided to allow replication by others?

Yes

Are the datasets clearly presented in a useable and accessible format?

Not applicable

Competing Interests: No competing interests were disclosed.

Reviewer Expertise: Burn wound healing and scarring at the basic science level and clinical

I confirm that I have read this submission and believe that I have an appropriate level of expertise to confirm that it is of an acceptable scientific standard.

Reviewer Report 09 February 2022

https://doi.org/10.3310/nihropenres.14350.r28461

© 2022 Zuijlen P. This is an open access peer review report distributed under the terms of the Creative Commons Attribution License, which permits unrestricted use, distribution, and reproduction in any medium, provided the original work is properly cited.

Paul P. M. van Zuijlen
[1] Burn Centre, Red Cross Hospital, Beverwijk, The Netherlands
[2] Department of Plastic, Reconstructive and Hand Surgery, Amsterdam Movement Sciences, Amsterdam UMC (location VUmc), Amsterdam, The Netherlands

This is clinically interesting and relevant randomised, controlled trial where the Pulsed Dye Laser and standard care are compared against the standard care alone.

My only suggestion would be to reconsider and extend the length of follow-up to make the outcome of the study more appropriate. The authors discuss the length of the 6-month follow-up nicely in the protocol section and they hypothesize that a shorter-term improvement would be sufficient to show that PDL 'flattens the curve of scar maturation'. This 6-month follow-up is clearly a compromise for several reasons (reliability of data and demonstration of effectiveness). But the control scars will likely catch up to some degree after six months. Whether this will happen and whether this is clinically relevant is currently not being investigated. A long-term follow-up of 12 months is therefore recommended.

Is the rationale for, and objectives of, the study clearly described?
Yes

Is the study design appropriate for the research question?
Partly

Are sufficient details of the methods provided to allow replication by others?
Yes

Are the datasets clearly presented in a useable and accessible format?
Yes

Competing Interests: No competing interests were disclosed.

Reviewer Expertise: Acute burns, Scarring, Reconstructive surgery, Tissue engineering, Value Based Health Care, Clinimetry

I confirm that I have read this submission and believe that I have an appropriate level of expertise to confirm that it is of an acceptable scientific standard.
