# Supplementary material for: Early Laser for Burn Scars (ELABS): protocol for a multi-centre randomised, controlled trial of both the effectiveness and cost-effectiveness of the treatment of hypertrophic burn scars with Pulsed Dye Laser and standard care compared to standard care alone
Source: NIHR Open Res. 2022 Jan 18;2:1. [Version 1] doi: 10.3310/nihropenres.13234.1 (PMC7612584; doi:10.3310/nihropenres.13234.1)
Supplement: Peer review history

# Reviewer report: Reviewer response for version 1

**DOI:** 10.3310/nihropenres.14350.r28465

**Published:** 2022-02-28

**Authors:** Mamta Shah

**Affiliations:** 1 Burns and Plastic Surgery Department, Royal Manchester Children's Hospital, Manchester, UK

The researchers present a well thought out and reasoned study protocol on the early use of LASER to minimise the morbidity of post-burn hypertrophic scarring. They define early as the period within 3 months of complete healing. Hypertrophic scarring following a burn injury is one of the most common causes of morbidity for burn patients resulting in functional and psychological consequences. There is emergent evidence to demonstrate that the use of PDL on post-burn hypertrophic scars (HBS) alleviates itching and reduces redness of the scars. 

 The basis of this randomised controlled trial is to ascertain if the use of PDL early in the trajectory of the post-burn hypertrophic scar process, prevents the scar from becoming red, lumpy, firm/hard and causing subsequent problems with function and symptoms such as itching as well as alleviating the existing symptoms and quality of the scar at start of treatment. 

 The researchers have involved patient group and discussed all aspects of the study with them. However, it is unclear how many of the 18 patients involved in this study design and discussion had already had laser treatment and how many had not? Was there an element of bias in the discussions from the group who had the PDL treatment if not equally distributed?

 The researchers have selected a period of within 3 months of healing of target site as a criteria and not from time of injury. That has to be commended as it is important to ensure the trajectory is from time to complete healing and not time of injury! It is also important that the final follow-up assessment is at least 3 months from time of last intervention and not 6 months from time of 1st treatment. Another point to note in patient selection, the researchers are selecting not only post-burn scars but also donor site scars for their study. These different mechanistic scars should not be included into one cohort as a collective due to the different mechanisms of injury and subsequent wound healing and scarring (Jabeen *et. al*; 2019 ^1^).  

 The researchers have stated that this study does not test the various PDL treatment settings/intensity and duration but that they have selected their regime from a review of literature: perhaps some clarification on how this was done would be useful. If the various centres participating in this study are already using PDL for management of their burn patients, how did they agree to the current protocol as the optimum one to use? 

 The primary and secondary objectives are well defined. However, the final assessment of 6 months i.e. 3 months from final treatment, will not allow the researchers to make any claims of long term impact on the post-burn hypertrophic scarring with regard to alteration of the trajectory or prevention of long term sequelae. They have explained their rationale but it would be prudent to include in their ethics design and study protocol a caveat that allows them to follow these patients long term if we are to get any insight into whether early LASER intervention precludes long term sequelae of HBS and indeed pushes the natural trajectory of scar maturation process to the left. Some or most of these patients, presumably will be followed up in the Consultants clinics and if so, would not be too cumbersome to assess them long term. It would be a shame to lose this opportunity of ensuring the long term impact of early intervention as it is crucial to the study "hypothesis". Furthermore, it will ascertain how long the effects of PDL last if not permanent.

Is the study design appropriate for the research question?

Yes

Is the rationale for, and objectives of, the study clearly described?

Yes

Are sufficient details of the methods provided to allow replication by others?

Yes

Are the datasets clearly presented in a useable and accessible format?

Not applicable

Reviewer Expertise:

Burn wound healing and scarring at the basic science level and clinical

I confirm that I have read this submission and believe that I have an appropriate level of expertise to confirm that it is of an acceptable scientific standard.

## References

[1] : Partial thickness wound: Does mechanism of injury influence healing?. *Burns* .45(3) : 10.1016/j.burns.2018.08.010 531-542 10.1016/j.burns.2018.08.010 30739729

# Response

**Published:** 2023-01-23

**Authors:** Mark Brewin

**Affiliations:** Salisbury NHS Foundation Trust, UK

Thanks Mamta for your well informed comments. We shall endeavour to answer each comment in turn. It is a valid point that you make in asking for the data of how many patients in the PPI group has PDL treatment.

 This data is valuable and may have been lost in one of the many edits of our documents. For the records, of the 18 attendees, there were 9 burn injury patients, all of whom had had laser treatment, 6 had received laser treatment for non-burns and 1 who has had treatment for a surgical scar. As for the within 3 months of healing regimen, we have tried to make it as early as possible while considering that newly healed scale may still be fragile. Any adverse events or effects of laser treatment this soon will be picked up in the adverse event data within the study protocol. It is understood that donor sites are different to burn injury sites and data is captured as to whether the study scar is burn site or donor site. To date, no donor sites have been chosen (at recruit 93 of 150 required).

 The laser settings were chosen after an extensive literature search (Brewin *et al* 2014), a service evaluation conducted at Salisbury between 2014-2018 and an agreement between principal investigators at all sites included in the trial. It is accepted that some burn scar may be of different colour and thickness but it was reckoned that as the laser treatment is starting early, the scars should be relatively thin (though admittedly somewhat thickened) and red in nature. This would indicate the need to use a superficial 0.5 ms pulse. Experience at our centre has shown best results when an outcome of strong purpura is achieved, thus the relatively aggressive energy fluences.

 We agree that the 6 month follow up may be insufficient to make long term assumptions and we have answered this point in Reviewer 1’s comments here. A plan is in writing to follow up these patients further and consent for this is given by the recruit at the enrolment stage of this study. It was always our intention to follow up long term but this was not possible within the NIHR Research for Patient Benefit grant time-frame.

# Reviewer report: Reviewer response for version 1

**DOI:** 10.3310/nihropenres.14350.r28461

**Published:** 2022-02-09

**Authors:** Paul P. M. van Zuijlen

**Affiliations:** 1 Burn Centre, Red Cross Hospital, Beverwijk, The Netherlands; 2 Department of Plastic, Reconstructive and Hand Surgery, Amsterdam Movement Sciences, Amsterdam UMC (location VUmc), Amsterdam, The Netherlands

This is clinically interesting and relevant randomised, controlled trial where the Pulsed Dye Laser and standard care are compared against the standard care alone.

 My only suggestion would be to reconsider and extend the length of follow-up to make the outcome of the study more appropriate. The authors discuss the length of the 6-month follow-up nicely in the protocol section and they hypothesize that a shorter-term improvement would be sufficient to show that PDL 'flattens the curve of scar maturation'. This 6-month follow-up is clearly a compromise for several reasons (reliability of data and demonstration of effectiveness). But the control scars will likely catch up to some degree after six months. Whether this will happen and whether this is clinically relevant is currently not being investigated. A long-term follow-up of 12 months is therefore recommended.

Is the study design appropriate for the research question?

Partly

Is the rationale for, and objectives of, the study clearly described?

Yes

Are sufficient details of the methods provided to allow replication by others?

Yes

Are the datasets clearly presented in a useable and accessible format?

Yes

Reviewer Expertise:

Acute burns, Scarring, Reconstructive surgery, Tissue engineering, Value Based Health Care, Clinimetry

I confirm that I have read this submission and believe that I have an appropriate level of expertise to confirm that it is of an acceptable scientific standard.

# Response

**Published:** 2023-01-23

**Authors:** Mark Brewin

**Affiliations:** Salisbury NHS Foundation Trust, UK

Thanks Paul for your comments on our trial. As you have surmised, the 6 month follow-up is a compromise due to several reasons. Firstly, the funding stream (NIHR RfPB) demands are for a 3 year trial period and this should include study set up as well as data analysis and write-up. This reduces the time period by a minimum of 6 months. In order to get sufficient recruits to power the study, we need a number of sites and a lengthy recruitment period (in this case 21 months). This prevents us from a lengthier follow-up period. In addition, a 6 month study period keeps the study attractive to potential recruits as some people are wary of committing to a longer follow-up than this. Finally attrition is a consideration as people will be lost to follow-up for longer follow-up periods. There is a plan afoot to further follow-up the patients to collect Patient POSAS scores and this may form an interesting PhD or MPhil project. The potential for follow-up was written into the study consent form so we can capture this data with the consent of the patient. This "extra" paper will be of huge interest.
